# Autochthonous transmission of dengue in Europe, with special attention to the Spanish context

**DOI:** 10.1017/S095026882610123X

**Published:** 2026-02-27

**Authors:** Marta Bononad Brugger, M. Adela Valero, María Morales-Suárez-Varela

**Affiliations:** 1 Research group in Social and Nutritional Epidemiology, Pharmacoepidemiology and Public Health, Department of Preventive Medicine and Public Health, Food Sciences, Toxicology and Forensic Medicine, Faculty of Pharmacy and Food Sciences, Universitat de València; 2 Department of Pharmacy and Pharmaceutical Technology and Parasitology, Faculty of Pharmacy and Food Sciences, Universitat de València; 3 Biomedical Research Center in Infectious Disease, (CIBERINFEC), Carlos III Health Institute; 4 Biomedical Research Center in Epidemiology and Public Health Network (CIBERESP), Carlos III Health Institute

**Keywords:** Aedes, autochthonous transmission, dengue, Europe, vector-borne disease

## Abstract

Autochthonous transmission of dengue in southern Europe has emerged as a growing public health concern, especially in regions such as Spain, due to the expansion of mosquito vector species, such as *Aedes albopictus* and *Aedes aegypti*, introduced into these regions. This article presents an overview of the situation based on the analysis of the different reports published by international and national health agencies, together with key scientific studies on autochthonous transmission of dengue in Europe and Spain. Through this work, the factors considered to be contributing or hypothesized drivers of the spread of the virus on the European continent, such as climate change, human mobility, and the proliferation of mosquito vectors, are described. It explores the cases of autochthonous transmission documented in several European countries and Spain. In addition, the surveillance protocols implemented by Spanish health authorities and the health responses to outbreaks in Spain are also examined. Finally, the risk of future transmission in Spain is assessed, and strategies are proposed to strengthen epidemiological surveillance, improve preparedness for possible outbreaks, and optimize vector-control policies in the context of global change.

## Introduction

Dengue is endemic in more than 100 countries, including most tropical and subtropical countries in Central America, the Caribbean and South America, Southeast Asia, the South Pacific and Western Pacific, the Eastern Mediterranean, Oceania, and sub-Saharan Africa [1]. A recent study estimates that worldwide, 5.6 billion people are at risk of infection with dengue and other arboviruses, and there are an estimated 390 million new cases of dengue infection annually, of which 96 million manifest clinically [2–4].

In the European Region, the last dengue epidemic occurred in Greece and Spain between 1927 and 1928, and since then, until 2010, all reported cases have been imported. In 2010, the first two autochthonous cases were reported in France and Croatia. The last autochthonous dengue outbreak on the island of Madeira started in October 2012. Although in all three locations the virus detected was serotype 1 (DENV-1) genotype III, molecular epidemiology studies show that these were three different reintroductions, from the Caribbean, India, and South America, respectively [5]. However, in Europe, autochthonous dengue cases have been increasing in recent years, with a marked increase in 2023 and 2024. European countries with confirmed autochthonous cases include Italy, France, Spain, Croatia, and Portugal (Madeira) [6–8]. In 2024, 308 cases were reported to the World Health Organization (WHO) from three European countries (France, Italy, and Spain) [2]. The autochthonous spread of the disease is strongly related to the presence of a suitable vector, an adequate pool of viraemic people, and suitable climatic conditions for both the survival of the vector and the development of the virus in the vector [9, 10]. The current conditions in Europe suit the local transmission of dengue fever, especially in southern Europe [10].

The mosquito species responsible in Europe is mainly *Aedes albopictus* (tiger mosquito), which is established and expanding in several European countries, such as Spain, France, Italy, and others. In addition, *Aedes aegypti*, which is more efficient in transmitting the virus [11] primarily due to its higher vectorial capacity and specific adaptation to human environments, is present in outermost regions of the EU, such as Madeira (Portugal) and Cyprus. *Ae. aegypti* generally shows equal or higher transmission potential than *Ae. albopictus* in experimental and field studies, though results are context dependent. *Ae. aegypti* often has higher realized transmission, while *Ae. albopictus* can contribute to persistence via vertical transmission [12–15]. Available field data show contrasting seasonal activity patterns, with *Ae. albopictus* maintenance in cooler months via vertical infection and *Ae. aegypti* prominence in warm rainy periods [13, 16].

**Figure 1.**
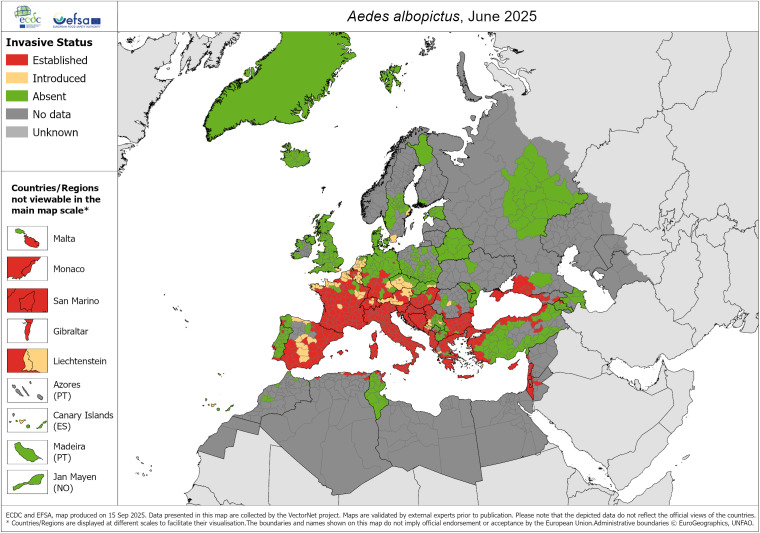
*A. albopictus* – current known distribution: June 2025. Source: European Centre for Disease Prevention and Control and European Food Safety Authority. Mosquito maps [internet]. Stockholm: ECDC; 2025. Available from: https://www.ecdc.europa.eu/en/publications-data/aedes-albopictus-current-known-distribution-june-2025.

**Figure 2.**
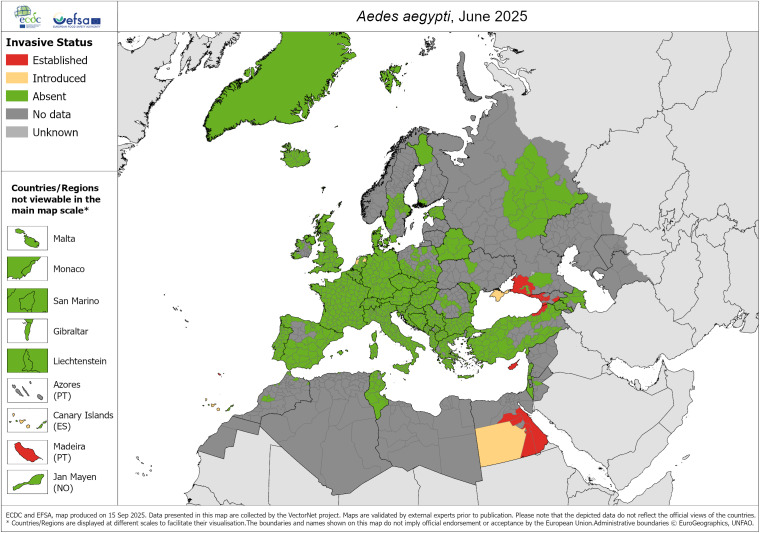
*A. aegypti* – current known distribution: June 2025. Source: European Centre for Disease Prevention and Control and European Food Safety Authority. Mosquito maps [internet]. Stockholm: ECDC; 2025. Available from: https://www.ecdc.europa.eu/en/publications-data/aedes-aegypti-current-known-distribution-june-2025.

**Figure 3.**
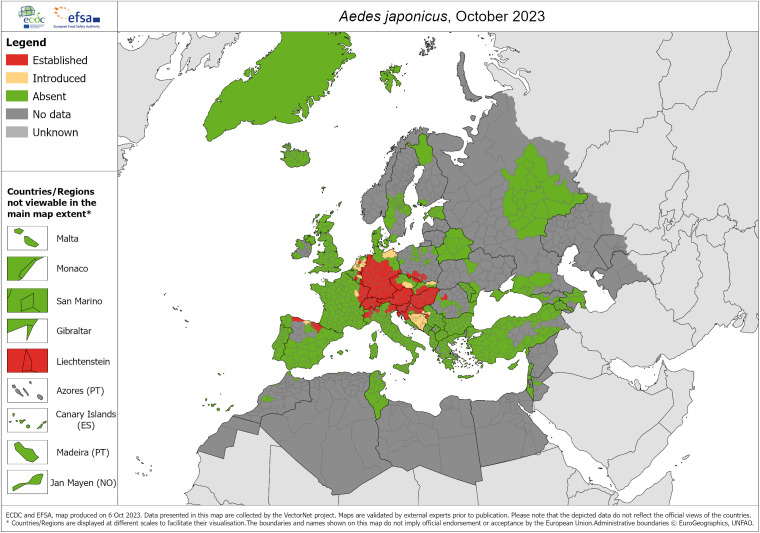
*Aedes japonicus* – current known distribution: October 2023. Source: European Centre for Disease Prevention and Control and European Food Safety Authority. Mosquito maps [internet]. Stockholm: ECDC; 2023. Available from: https://www.ecdc.europa.eu/en/publications-data/aedes-japonicus-current-known-distribution-october-2023.

Specifically in Spain, the invasive vector species of dengue widespread in the Iberian Peninsula are *Ae. albopictus* and, in some northern areas, *Aedes japonicus* [17, 18]. However, *Ae. aegypti* has been sporadically detected in the Canary Islands, but it is not considered an established species and does not have permanent populations in the Spanish archipelago. Only sporadic colonizations have been recorded, and entomological surveillance is still active to prevent its establishment. The latest known distributions in Europe for the three mosquito species are shown in Figures 1–3.

This increase in autochthonous cases is mainly attributed, by the existing available literature, to climatic factors, such as global warming that favours the expansion and activity of mosquito vectors, and to the increasing circulation of the dengue virus imported by travellers. Although autochthonous transmission is already a reality, it is considered that a formal endemic has not yet been established on the continent. However, the European scientific and health community is alert to the risk that dengue may become endemic in some regions in future.

The probability of this disease appearing in Spain will depend mainly on the virus being introduced into an area where the appropriate vectors are established, their density, and interaction with the susceptible population. Dengue virus has been detected on one occasion in an *Ae. albopictus* specimen captured in Catalonia in the environment of an imported case, demonstrating the capacity of local vectors to become infected with this virus. In addition, it will be influenced by sociodemographic, ecological, economic, and cultural factors of the environment in which it develops [5]. In this regard, it should be remembered that Spain has a large exchange of travellers with America and other regions with indigenous transmission of arbovirosis. Therefore, the risk of introducing cases of dengue associated with travel is very high, and in territories where *Ae. albopictus* is established, the probability of the appearance of associated autochthonous cases is moderate, while in territories with no vector detection and occasional and recent detection, it is considered very low. To avoid local transmission of arbovirus, it is highly important to implement or maintain the measures described in the Plan for the Prevention, Surveillance and Control of Vector-Borne Diseases [19].

The activity of *Ae. albopictus* varies throughout the year, depending on the environmental conditions of humidity and temperature and the population density of the area where it is established. Spain meets the necessary conditions for virus circulation and, therefore, the occurrence of autochthonous cases of dengue: the presence of a competent vector (*Ae. albopictus*), a significant flow of travellers from endemic areas or areas with active transmission of the disease that can introduce the virus, and climatic conditions suitable for maintaining the biological cycle of the virus once it is introduced. The most likely period in which autochthonous cases may appear is from May to October–November, due to higher vector activity and density, although in some parts of Spain, *Ae. albopictus* activity has been detected even in December [19, 20].

Most arboviral infections transmitted by *Ae. albopictus* have mild and self-limiting symptoms in most cases [20]. The nonspecific nature of the symptoms and the possible existence of asymptomatic cases may delay healthcare seeking and hinder timely diagnosis by healthcare professionals, particularly when clinical suspicion is low. Autochthonous cases of severe or haemorrhagic dengue, characteristic of endemic areas, are not expected to occur in Spain or the European WHO region.

Taking into account the presence of the competent vector, the fact that the Spanish population is susceptible to the disease, and the sporadic introduction of the virus with the arrival of imported human cases, autochthonous transmission of dengue, although unusual, is a possible and expected event, as has occurred in recent years in other countries in continental Europe, such as France, Italy, and Croatia [8, 21].

The aim of this paper is to review the autochthonous cases of dengue reported in Europe during the period 2012 to 2025. This analysis aims to provide an updated overview of the epidemiological situation of dengue in the continent, with particular focus on the Spanish context and on the factors that favour local transmission in countries traditionally free from the disease.

In the EU, dengue case classification for surveillance purposes is governed by the harmonized definitions included in the annex of Commission Implementing Decision (EU) 2018/945 and by the operational case lists issued by ECDC, which ensure comparability across Member States (possible/probable/confirmed cases based on clinical, epidemiological, and laboratory criteria). In Spain, the national surveillance protocol of RENAVE, according to the European Commission, specifies that a case is considered confirmed when at least one of the following laboratory criteria is met: (i) isolation of dengue virus from a clinical specimen; (ii) detection of viral nucleic acid in a clinical sample; (iii) detection of viral antigen; (iv) seroconversion or a fourfold rise in dengue-specific antibody titres in paired serum samples collected approximately 15 days apart; or (v) detection of dengue-virus-specific IgM antibodies confirmed by neutralization. Conversely, a case is classified as probable when dengue-specific IgM antibodies are detected in a single serum sample without neutralization confirmation [22].

## Methods

### Study design and methodological framework

This study is a scoping review with a descriptive surveillance analysis of autochthonous dengue transmission in Europe, with a particular focus on Spain, conducted in accordance with the PRISMA-ScR guidelines to ensure transparency in the search strategy and source selection. The aim of the review was to document and synthesize the occurrence of autochthonous cases and clusters, describe their spatial and temporal distribution, and characterize the response and control measures implemented.

### Information sources and prioritization

For consolidated data on confirmed autochthonous dengue cases in Europe, the ECDC Surveillance Atlas of Infectious Diseases was prioritized as the primary source (harmonized and validated data up to 2024). For very recent events (2025), non-consolidated reports were used, including ECDC Rapid Risk Assessments/Threat Reports and CCAES (MISAN) alerts and reports, as well as information from Eurosurveillance, WHO Disease Outbreak News, and bulletins issued by national and regional ministries of health and surveillance networks. Entomological information (presence/distribution of *Aedes* spp.) was obtained from ECDC-VectorNet and ECDC vector distribution maps. Scientific literature retrieved from PubMed/MEDLINE, Scopus, Embase, and Web of Science was used to complement, contextualize, and confirm epidemiological details and response measures. No non-public surveillance data were used, and all underlying datasets can be accessed by other researchers.

### Priority rule for discrepancies

1. ECDC Atlas (years with complete consolidation: up to 2024).
2. ECDC RRAs/Threat Reports and Eurosurveillance (recent and still-consolidating events).
3. CCAES/MISAN and national/regional authorities (for Spain and subnational details).
4. Other official sources (WHO).

Conflicting figures were resolved by adopting the values from the source with the highest priority.

### Search strategy

The search strategy aimed to identify documents published between 1 October 2012 and 5 October 2025, the date of the last update. Sources in Spanish and English were included, and when necessary for national reports, documents in French, Italian, and Portuguese were also consulted, with translation of titles and abstracts when required.

The search encompassed a wide range of sources. Institutional websites and databases were consulted first, including the ECDC Atlas, the Annual Epidemiological Reports on dengue published by the ECDC, Threat Reports, Rapid Risk Assessments, and the daily and weekly alerts and reports issued by CCAES/MISAN. Communications from the WHO through Disease Outbreak News, websites of European national health ministries and surveillance networks, and entomological information from VectorNet – particularly regarding the distribution of *Aedes* species – were also reviewed.

Searches were additionally conducted in bibliographic databases, specifically PubMed/MEDLINE and Scopus, and, when institutionally accessible, also Embase and Web of Science, to identify relevant scientific literature for the study period and geographical scope. Finally, grey literature from national and regional surveillance bulletins and institutional repositories was included to ensure comprehensive coverage of official and complementary sources related to autochthonous dengue transmission in Europe and Spain.

### Search strings

PubMed:

dengue

AND (autochthonous OR “local transmission”)

AND (Europe OR Spain OR “European Union” OR EU)

AND ("2012/10/01"[Date - Publication]: “2025/02/15”[Date - Publication])

Scopus:

TITLE-ABS-KEY(dengue AND (autochthonous OR “local transmission”)

AND (Europe OR Spain OR “European Union” OR EU))

AND PUBYEAR > 2011 AND PUBYEAR < 2026

Web of Science:

TS=(dengue AND (autochthonous OR “local transmission”)

AND (Europe OR Spain OR “European Union” OR EU))

Google Scholar:

dengue “autochthonous” OR “local transmission” Europe Spain “European Union” EU

Institutional searches:

dengue AND (autochthonous OR “local transmission”) AND (Europe OR Spain)

All references were managed using a reference management tool with automated deduplication and manual verification.

### Definitions and geographical scope

Autochthonous dengue transmission was defined as a confirmed case with no travel history to an endemic area in the preceding 14 days and/or epidemiological linkage to a locally acquired infection. Case definitions and laboratory confirmation criteria were adopted from ECDC and MISAN guidelines (RT-PCR, NS1, IgM/IgG seroconversion according to standards applicable during each period).

The geographical scope covered EU/EEA countries (as defined annually by the ECDC) and Spain, at national and subnational levels (autonomous communities and, when available, provinces/municipalities). Spanish archipelagos (Balearic Islands and Canary Islands) were included. Madeira (Portugal) was considered within the European analysis.

### Eligibility criteria

Inclusion criteria:

- Reports/series/articles explicitly mentioning autochthonous dengue cases in the EU/EEA and/or Spain.
- Documents describing the presence/distribution of *Aedes* spp. and their relationship with local transmission.
- Reports describing public health responses, control measures, or risk management for autochthonous outbreaks.
- Source types: ECDC Atlas/AER/Threat Reports, CCAES/MISAN, WHO-DON, national/regional bulletins, Eurosurveillance, and peer-reviewed articles.

Exclusion criteria:

- Reports or cases exclusively imported (travel-related) with no evidence of local transmission.
- Commentaries/editorials without data; media reports lacking official confirmation.
- Purely predictive modelling without observational data.
- Non-European regions.

### Quality assessment and risk of bias

Quality and risk of bias were evaluated using different tools depending on the type of document. For institutional reports and grey literature (ECDC, CCAES, national/regional bulletins), the assessment focused on authority, accuracy, coverage, objectivity, date, and relevance. For observational studies, such as case series or prevalence studies, specific criteria assessing methodological validity and design quality were applied. When a single event was described across multiple sources, consistency and verifiability were assessed by examining coherence across documents, traceability of case definitions, diagnostic methods used, and availability of denominators and additional epidemiological details. Quality assessments were incorporated into the synthesis of findings, marking data as preliminary or low confidence when appropriate. Official sources were not systematically excluded unless they presented critical deficiencies, such as the absence of a case definition, lack of diagnostic confirmation, or manifestly inconsistent data.

### Limitations

This study has several limitations that must be considered when interpreting the results. First, there is marked heterogeneity in case definitions and surveillance and diagnostic practices across European countries and throughout the study period, including changes in confirmation and notification criteria that may affect data comparability. In addition, some of the consulted reports – particularly ECDC Rapid Risk Assessments and CCAES reports – are provisional and may be updated; thus, a distinction was made between consolidated information available in the ECDC Atlas up to 2024 and preliminary data corresponding to events reported from 2025 onward.

Under-recognition and under-diagnosis of dengue also represent significant limitations, given the high proportion of asymptomatic or mildly symptomatic cases and the variability in diagnostic capacity across regions. This issue is further complicated by possible serological cross-reactivity with other arboviruses such as chikungunya or Zika, or flaviviruses such as West Nile virus, which can hinder accurate diagnosis in the absence of molecular confirmation.

Additional limitations arise from availability bias in grey literature and uneven subnational coverage, which can influence the level of detail and completeness of reported information. Moreover, the use of vector distribution maps entails interpretative constraints, as entomological presence does not imply transmission, and both mosquito density and vector competence may vary considerably within areas classified as positive. Finally, due to heterogeneity in sources, study designs, and levels of data consolidation, a meta-analysis was not feasible; instead, a descriptive synthesis was performed to coherently integrate the available evidence.

## Results and discussion

### Recent autochthonous dengue cases

In Europe, the last dengue virus epidemic occurred in 1927. From that time until 2010, all diagnosed cases of dengue were imported cases. The first cases of recent autochthonous transmission in Europe were reported in 2010 in France (two cases) and Croatia (15 cases) [23, 24]. Sporadic cases have been detected in France in 2013, 2014, 2015, and 2018, without sustained transmission during the winter period [25–27].

Outside continental Europe, two substantial dengue outbreaks have been reported: the 2012 outbreak in Madeira (Portugal), involving more than 2,000 cases associated with *Ae. aegypti* [28, 29]; and the 2018–2021 outbreak in Réunion (France), with over 30,000 cases associated with *Ae. albopictus* [30, 31].

The following subsections offer a detailed overview of the epidemiological status of dengue in selected European countries.

#### Portugal

The *Ae. aegypti* species was first detected in Funchal, the capital of Madeira Island, in 2004, and in October 2012, the island reported its first autochthonous case of dengue DENV-1 through the European Early Warning and Response System (EWRS). The circulating serotype detected was similar to the one circulating at the time in Venezuela, Colombia, and Brazil [32–34]. This was the first autochthonous dengue outbreak recorded in Europe [32–35].

A dengue outbreak was declared in Madeira following the confirmation of two cases in Funchal and Caniço. Since then, a total of 1,891 confirmed cases were recorded that year (out of 2,187 probable cases reported), 100 of which required hospital admission. The clinical manifestations of most cases were mild, even asymptomatic. A further 81 cases were reported in 13 other European countries in travellers returning from Madeira (11 cases in Portugal and 70 in other European countries) [32, 33]. In the area around Funchal, rates of 146–524 cases per 10,000 inhabitants were reached. The characteristics of the island favoured the occurrence of the outbreak (high population density, climatic and environmental conditions that prolong the life of the vector and, therefore, the probability of local transmission).

The importance of this epidemic is demonstrated by three main reasons: it was the first sustained autochthonous transmission of dengue in the EU since the 1920s; its size; and the rapid time course of the epidemic, peaking within one month of the official report of the first case in October, with a sharp decline thereafter, with no cases reported after 3 March 2013.

To understand the complexities of this outbreak, Lourenco and Recker developed an entoepidemiological mathematical model to explore the ecological conditions and transmission dynamics [34, 35]. Their findings suggest that the virus was introduced more than a month before cases were reported (in August 2012), during the peak period of air travel. In their model, asymptomatic circulation occurred before the first two autochthonous cases were reported in October, circulating the virus in the population. Furthermore, their findings indicate that transmission dynamics and the eventual epidemic were predominantly driven by the influence of temperature on the mosquito life cycle (incubation period, mosquito mortality, and aquatic development rates). In addition, because the summer of 2012 was slightly warmer than usual, the epidemic potential and probability of invasion were higher compared to typical temperatures at that time on the island, which may explain the success of the dengue virus in that particular year. The seasonal drop in autumn temperatures led to a reduction in vector capacity and effectively halted the spread of the virus. The temperature in the autumn months reduced the number of adult mosquitoes, but more importantly, it fell below the critical threshold where the incubation period is shorter than the average lifespan of mosquitoes and subsequent transmission to humans. This effectively reduced vectorial capacity and halted viral spread, leading to a significant decrease in dengue cases [34, 35].

It is also important to take into account seasonal variations in temperature in the possible epidemic dynamics on Madeira Island. The influence of seasonality must be emphasized, as Madeira Island has a variety of contrasting bioclimates due to its heterogeneous landscape and strong influence of the Gulf Stream and currents from the Canary Islands, in addition to the variation between the different areas of the island in terms of solar exposure, humidity, and temperature. The southern coastal regions of the island (including Funchal), at low altitudes, have higher annual temperatures compared to the northern coastal regions or the interior, regions with higher altitudes and lower annual temperatures. Further introductions of dengue are likely to occur on the island, and local transmission could occur if the virus is introduced, given the presence of the vector and favourable environmental conditions [34, 35].

In the aforementioned study [34, 35], it was shown that epidemic dynamics are strongly influenced by variation in the timing of dengue virus introduction, seasonal temperature, transmission rates, mortality rate, and mosquito population. The sensitivity analysis of the model provides insight into the relative importance of these parameters and their interactive effects as drivers of an epidemic. These results can be a useful guide in the development of effective local dengue control and mitigation strategies on Madeira Island [34, 35].

Using key estimates, together with local climatic data, the study proposes that dengue endemicity on this island is unlikely, but there is a high potential for future epidemic outbreaks when the virus is introduced between May and August, a period when detection of imported cases is crucial for public health in Madeira [34, 35].

As of the beginning of 2025, the island of Madeira had not reported any dengue cases since the 2012 outbreak. However, there was a high risk that virus introduction could lead to another autochthonous outbreak given the presence of the vector and the favourable environmental conditions for dengue transmission, especially in summer and autumn [35].

On 7 February 2025, the National Reference Laboratory for Entomology reported the detection of DEN-2 in a pond with nine *Ae. aegypti* mosquitoes in Madeira [36]. The mosquitoes were collected between 13 January 2025 and 24 January 2025, in Funchal, in the context of active surveillance in an area where a case with symptoms compatible with dengue and no history of travel had been investigated in early January. This was a suspected case where the diagnosis was not confirmed at the time but had a history of having received visitors from a dengue-endemic country, who were staying in the same residential area. The Madeira health authorities implemented response measures, including strengthening entomological and epidemiological surveillance, with active detection. On 18 February 2025, new laboratory results were available retrospectively confirming the case with onset of symptoms in early January 2025, as well as a second case, a cohabitant of the first case [37] (see Table 1).

**Table 1.** Summary of dengue cases in Portugal

Location	Dates/interval	Cases	Source
Madeira Island – Funchal & Caniço (Portugal)	Oct 2012–3 Mar 2013	1,891 confirmed (2,187 probable); 100 hospitalizations	[27–30]
Europe (13 countries; travel-associated from Madeira)	Oct 2012–Mar 2013	81 travel-associated cases (11 in Portugal; 70 in other EU countries)	[27, 28]
Madeira Island – Funchal (Portugal)	Early Jan 2025 (onset); confirmed 18 Feb 2025	2 autochthonous cases	[32]

#### France

Autochthonous outbreaks of dengue have been detected in France since 2010 [38]. On 24 April 2018, France reported a significant increase in dengue cases in Reunion Island since the beginning of 2018 [39]. As of 17 April 2018, a total of 1,388 confirmed cases were reported, compared to 100 cases reported during 2017. The circulating serotype was mostly serotype 2 (DENV-2).

From 1 May to 30 November each year, Santé publique France coordinates enhanced seasonal surveillance of dengue in metropolitan departments colonized by the mosquito vector, *Ae. albopictus.*

During the 2019 enhanced surveillance period, there were nine autochthonous cases of dengue reported in metropolitan France: an outbreak of two autochthonous cases of dengue in the Rhône department and an outbreak of seven autochthonous cases in the Alpes-Maritimes department [40].

In the 2020 enhanced surveillance period, six autochthonous dengue outbreaks (13 cases) were identified [41]. In Provence-Alpes-Côte d’Azur, three separate outbreaks were identified: first, an outbreak of three autochthonous dengue cases with onset dates between 1 August 2020 and 11 August 2020 in La Croix-Valmer (Var); another cluster of five cases in Nice with onset dates between 11 August 2020 and 4 September 2020 [41]; finally, an outbreak of two cases with onset dates between 19 September 2020 and 4 October 2020 in Saint Laurent du Var [41]. Meanwhile, in Occitanie, three isolated autochthonous cases were also reported: one in the Hérault department with an onset date on 17 July 2020, one contracted in Hérault or Gard, with an onset date of 31 August 2020, and one in Gard, with an onset date of 3 September 2020 [41].

On 26 July 2021, the French health authorities reported the first case of autochthonous dengue for the 2021 season in the Var area [42]. On 16 August 2021, they reported an autochthonous dengue case in Toulon. However, on 17 August 2021, the case was ruled out due to the results of additional tests performed, which included negative serology and PCR, in addition to the detection of autoimmune disease in the patient, indicating a false-positive AgNS1 [42]. Another autochthonous case of dengue was identified in Montpellier on 5 November 2021. No further cases were identified during this surveillance period [42].

In 2022, France reported nine outbreaks involving 65 cases of autochthonous dengue [38].

In the Corsica region, there was an outbreak of two autochthonous cases whose symptoms began in mid-September. Three outbreaks totalling 51 cases were reported in the Provence-Alpes-Côte d’Azur region. In the Var department, an outbreak of seven cases of dengue was reported in Fayence, with symptoms appearing between late June and late July. In the Alpes-Maritimes department, an outbreak of 34 cases in the neighbouring municipalities of Saint Jeannet (22 cases), Gattières (11 cases), and La Gaude (one case) with symptoms beginning between early August and late September and an outbreak of ten cases in the municipalities of Saint Laurent du Var (nine cases) and Cagnes sur Mer (one case exposed in St Laurent du Var) with symptoms beginning between mid-August and mid-September were recorded. These two Alpes-Maritimes outbreaks are distinct because they are caused by different serotypes of the virus (DENV-1 and serotype 3 (DENV-3)).

In the Occitania region, there were five outbreaks totalling 12 cases. In the Pyrénées-Orientales department, in Perpignan, there was one locally acquired case of dengue, with symptoms appearing in mid-June. In the Hautes-Pyrénées, there was an outbreak of four cases in the municipalities of Andrest (three cases) and Rabastens (one case), with symptoms beginning between mid-July and the end of August. In Haute-Garonne, in La Salvetat Saint Gilles, there was an outbreak of four cases of dengue in the same household, with symptoms beginning in the last two weeks of August. In the Tarn-et-Garonne department, one case was likely infected during a stay in Montauban at the end of August. In Haute-Garonne, there was an outbreak of two cases in Toulouse, occurring in the same household, with symptoms beginning in the second half of September.

In the Andrest outbreak, the French health authorities reported a case of dengue fever in a 16-year-old female with a history of travel to Catalonia from 4 August 2022 to 21 August 2022 and mosquito bites. The case started experiencing symptoms on 17 August 2022, with generalized pain and fever, while still in Catalonia, and was hospitalized on 26 August 2022, back in the Occitanie region due to persistent symptoms. In Catalonia, no related cases were reported in the places where the case had travelled.

In 2023, France reported nine outbreaks involving a total of 45 autochthonous cases [43].

In the Provence-Alpes-Côte d’Azur region, outbreaks were reported in the Bouches-du-Rhône and Alpes-Maritimes departments. In Bouches-du-Rhône, an outbreak of four dengue cases was identified in Gardanne, with the onset of symptoms occurring between the second half of July and mid-August, and a cluster of ten cases was identified in Boulbon, with the onset of symptoms occurring between the end of July and the end of September. In the Alpes-Maritimes department, an outbreak of two cases was identified in Nice, with the onset of symptoms occurring between early August and mid-September, and another case was identified in Antibes, with the onset of symptoms occurring at the end of September.

Three outbreaks were reported in the Occitania region: a cluster of 11 cases around Perpignan in the Pyrénées-Orientales department, with the onset of symptoms occurring between late July and mid-August; a cluster of nine cases in Gagnières in the Gard department, with onset dates between late August and late September; and a cluster of three cases in Montpellier in the Hérault department, with symptoms appearing between mid-September and late September.

The remaining two outbreaks were reported in the Auvergne Rhône-Alpes and Ile-de-France regions. In Auvergne Rhône-Alpes, an outbreak of two cases was identified in Bourg-lès-Valence in the Drôme department, with symptoms appearing between late August and mid-September, while in Ile-de-France, an outbreak of three cases was identified in the Limeil-Brévannes department, with symptoms appearing in September.

Thanks to epidemiological investigations, the imported case responsible for the transmission was identified in three of the nine households investigated. In two households, the primary imported case had travelled to Martinique, and in one household, the primary imported case had travelled to Guadeloupe. The maximum distance between the places of residence of cases in the same transmission cluster did not exceed 950 m. For seven of the nine autochthonous clusters, the dengue virus serotype was identified. It was DENV-2 for four clusters, DENV-1 for two clusters, and DENV-3 for one cluster.

The provisional results for the 2024 season show 11 local dengue transmission outbreaks totalling 83 cases [44]. This is the highest number of outbreaks and autochthonous cases identified since enhanced surveillance was introduced in 2006. These outbreaks occurred in Provence-Alpes-Côte d’Azur (76 cases), Occitania (five cases), and Auvergne-Rhône-Alpes (two cases) regions. The first case began showing symptoms on 17 June 2024, in the Occitania region, and was the first case of autochthonous dengue reported in Europe in 2024 (see Table 2).

**Table 2.** Summary of dengue cases in France

Location	Dates/interval	Cases	Source
Réunion Island (France)	Jan–17 Apr 2018	1,388	[34]
Metropolitan France – Rhône department	May–Nov 2019 (enhanced surveillance)	2	[35]
Metropolitan France – Alpes-Maritimes department	May–Nov 2019 (enhanced surveillance)	7	[35]
Provence-Alpes-Côte d’Azur – Var (La Croix-Valmer)	1–11 Aug 2020	3	[36]
Provence-Alpes-Côte d’Azur – Alpes-Maritimes (Nice)	11 Aug–4 Sep 2020	5	[36]
Provence-Alpes-Côte d’Azur – Alpes-Maritimes (Saint-Laurent-du-Var)	19 Sep–4 Oct 2020	2	[36]
Occitania – Hérault department	17 Jul 2020	1	[36]
Occitania – Hérault or Gard (probable)	31 Aug 2020	1	[36]
Occitania – Gard department	3 Sep 2020	1	[36]
Provence-Alpes-Côte d’Azur – Var area	26 Jul 2021 (first local case of the season)	1	[37]
Occitania – Hérault (Montpellier)	5 Nov 2021	1	[37]
Corsica region	Mid-Sep 2022	2	[33]
Provence-Alpes-Côte d’Azur – Var (Fayence)	Late Jun–Late Jul 2022	7	[33]
Provence-Alpes-Côte d’Azur – Alpes-Maritimes (Saint-Jean net, Gattières, La Gaude)	Early Aug–Late Sep 2022	34	[33]
Provence-Alpes-Côte d’Azur – Alpes-Maritimes (Saint-Laurent-du-Var, Cagnes-sur-Mer)	Mid-Aug–Mid-Sep 2022	10	[33]
Occitania – Pyrénées-Orientales (Perpignan)	Mid-Jun 2022	1	[33]
Occitania – Hautes-Pyrénées (Andrest, Rabastens)	Mid-Jul–End Aug 2022	4	[33]
Occitania – Haute-Garonne (La Salvetat-Saint-Gilles)	Late Aug 2022	4	[33]
Occitania – Tarn-et-Garonne (Montauban)	End Aug 2022	1	[33]
Occitania – Haute-Garonne (Toulouse)	Second half of Sep 2022	2	[33]
Provence-Alpes-Côte d’Azur – Bouches-du-Rhône (Gardanne)	Second half of Jul–Mid-Aug 2023	4	[38]
Provence-Alpes-Côte d’Azur – Bouches-du-Rhône (Boulbon)	End Jul–End Sep 2023	10	[38]
Provence-Alpes-Côte d’Azur – Alpes-Maritimes (Nice)	Early Aug–Mid-Sep 2023	2	[38]
Provence-Alpes-Côte d’Azur – Alpes-Maritimes (Antibes)	End Sep 2023	1	[38]
Occitania – Pyrénées-Orientales (around Perpignan)	Late Jul–Mid-Aug 2023	11	[38]
Occitania – Gard (Gagnières)	Late Aug–Late Sep 2023	9	[38]
Occitania – Hérault (Montpellier)	Mid–Late Sep 2023	3	[38]
Auvergne-Rhône-Alpes – Drôme (Bourg-lès-Valence)	Late Aug–Mid-Sep 2023	2	[38]
Île-de-France – Val-de-Marne (Limeil-Brévannes)	Sep 2023	3	[38]
Provence-Alpes-Côte d’Azur (region)	Season 2024 (provisional)	76	[39]
Occitania (region)	Season 2024 (provisional); first onset 17 Jun 2024	5	[39]
Auvergne-Rhône-Alpes (region)	Season 2024 (provisional)	2	[39]

#### Germany

On 1 February 2023, the Robert Koch Institute of Germany reported to the National Epidemiology Centre of the Carlos III Institute the detection of two cases of dengue (one confirmed and one probable), along with four cases compatible with epidemiological link, in residents of Germany with a history of travel to the Balearic Islands, specifically to Ibiza, during the incubation period [45, 46].

The confirmed case was a 27-year-old woman who travelled to Ibiza between 23 August 2022 and 30 August 2022. She stayed at a friend’s house with her partner, her 14-month-old daughter, and two adult friends. She started with symptoms compatible with dengue (fever, joint pain, and rash) on 31 August 2022. Her partner and daughter also started symptoms on 31 August 2022, but only the woman was tested for dengue, and dengue was confirmed by the detection of specific IgM antibodies and viral antigen. The diagnosis was confirmed in Germany on 8 September 2022, by detection of dengue-NS-1-Ag antigen together with positive IgM and negative IgG serology. On the same dates, her daughter presented with fever and nasal discharge, and her partner presented with sore throat, nasal discharge, and joint pain. Diagnostic tests were not performed in either of these two cases.

The probable case was a 37-year-old woman who travelled to Ibiza, to the same locality as the group described above, between 6 October 2022 and 13 October 2022, together with her partner and nine-year-old son and three other families. On 13 October 2022, she started with symptoms (fever, headache, muscle and joint pain, retro-orbital pain, and rash). The diagnosis of dengue was made by the detection of IgM antibodies. Serological diagnosis was made on 17 October 2022 in Germany, and IgM was positive without further determination. Her son also presented symptoms compatible with dengue a day before her, on 12 October 2022, and her partner on 15 October 2022, but in both cases, it was mild, and they did not consult health services, so in neither of these cases were diagnostic tests performed. On the other hand, none of their friends had symptoms compatible with dengue [45].

The Health Alerts and Emergencies Centre communicated the information received by Germany to the Epidemiology Service of the General Directorate of Public Health and Participation, Ministry of Health and Consumption of the Government of the Balearic Islands. Thanks to surveillance of the imported cases in Germany, the National Epidemiology Centre and the Epidemiology Service of the Balearic Islands identified the existence of a probable index case, imported from Mexico, who started symptoms on 11 August 2022 and stayed in Ibiza between 11 August 2022 and 31 August 2022, in the same locality as the cases detected by Germany [45].

The imported case, usually residing in Madrid, travelled from Mexico (27 July 2022 to 10 August 2022) and from there travelled to Ibiza and stayed in the same locality as the German cases described, between 11 August 2022 and 31 August 2022. On 1 August 2022, he started with symptoms (fever, severe headache, joint and muscle pain, nausea, and vomiting) and analytical alterations. On 1 September 2022, a diagnosis of dengue was made by positive IgM serology (see Table 3).

**Table 3.** Summary of dengue cases in Germany

Location	Dates/Interval	Cases	Source
Ibiza, Balearic Islands (Spain) – Group 1	Travel: 23–30 Aug 2022; symptom onset: 31 Aug 2022	1 travel-associated dengue case (confirmed, German resident); 2 symptomatic contacts untested	[40, 41]
Ibiza, Balearic Islands (Spain) – Group 2	Travel: 6–13 Oct 2022; symptom onset: 12–15 Oct 2022	1 travel-associated dengue case (probable, German resident); 2 symptomatic contacts untested; no symptoms in accompanying friends	[40]
Ibiza, Balearic Islands (Spain) – Probable index (imported from Mexico)	Stayed in Ibiza: 11–31 Aug 2022; symptom onset: 1 Aug 2022; dengue diagnosis: 1 Sep 2022	1 imported index case (resides in Madrid; travel to Mexico 27 Jul–10 Aug 2022)	[40]

#### Italy

*Ae. albopictus* is established in Italy, and autochthonous dengue cases have been reported since August 2020 in the Veneto region, where one case was reported [47].

In 2023, Italy notified 82 autochthonous dengue cases to the European Commission [47]. These cases refer to four unrelated episodes of transmission in the province of Lodi (41 confirmed cases), in the province of Latina (two cases), and in the province of Rome (38 cases with exposure in different parts of the metropolitan city of Rome and one case in Anzio, for which investigations are underway to verify any epidemiological links).

In 2024, 238 autochthonous confirmed cases of dengue fever were reported to the national surveillance system [47]. The largest outbreak, with 138 confirmed cases (data up to 28 October 2024), was observed in a municipality in the Marche region [48]. Sporadic cases and more limited outbreaks were reported in Lombardy, Veneto, Emilia-Romagna, Tuscany, Marche, and Abruzzo.

From 1 January to 23 September 2025, there were four confirmed autochthonous cases of dengue. Two separate events of local transmission of the dengue virus were identified in two regions, Emilia-Romagna and Veneto, one of which was a sporadic case and the other an outbreak (see Table 4).

**Table 4.** Summary of dengue cases in Italy

Location	Dates/Interval	Cases	Source
Italy – Veneto region	Aug 2020	1 autochthonous case	[42]
Italy – Lodi province	2023	41 confirmed autochthonous cases	[42]
Italy – Latina province	2023	2 autochthonous cases	[42]
Italy – Rome metropolitan area & Anzio	2023	38 cases in Rome +1 case in Anzio (epidemiological link under investigation)	[42]
Italy – Marche region (largest outbreak)	1 Jan–26 Nov 2024	138 confirmed cases	[43]
Italy – Lombardy, Veneto, Emilia-Romagna, Tuscany, Marche, Abruzzo	1 Jan–26 Nov 2024	Sporadic and limited outbreaks (total Italy: 213 cases)	[42]
Italy – Emilia-Romagna region	1 Jan–23 Sep 2025	1 local transmission event (part of 4 total cases in Italy)	[42]
Italy – Veneto region	1 Jan–23 Sep 2025	1 outbreak (part of 4 total cases in Italy)	[42]

#### Spain

In Spain, dengue has been a ‘notifiable’ or ‘compulsory declaration’ disease since 2015 [19]. Until 2018, only imported cases of dengue fever had been detected [19]. The evolution of the number of reported cases from 2016 to 2024 has been variable, with an average of 385 cases per year, a marked reduction in 2020 and 2021 when the minimum was reached (n = 50), and an increase in 2023 and 2024, years in which the highest numbers for the period were reached (615 in 2023 and 1,119 in 2024) [19]. Of the imported dengue cases with a known place of infection from 2016 to 2023, the proportion of cases originating in the Americas has been much higher than that of cases originating in other regions (1,423; 63% of cases); of these, most cases originated in the Caribbean and South America, with Cuba and the Dominican Republic being the most frequent countries of infection [8, 19, 21]. Of the imported cases with a known place of infection in 2024 (1,111; 99.8%), the most frequent region was Latin America, with 803 cases (72.3%), followed by Southeast Asia, with 152 (13.7%) [19].

In October 2018, autochthonous cases of dengue fever were reported for the first time in Spain, in people who had been in Andalusia, the Region of Murcia, and Catalonia at the probable time of transmission [8, 21]. This was the first time that autochthonous transmission of dengue was detected in Spain since the beginning of the 20th century [49]. Cases detected in neighbouring countries had already demonstrated that local transmission of this disease is possible in areas of Europe where some of the competent vectors are present.

These were three people belonging to the same family who were together outside their usual municipalities of residence, first in a holiday home in a municipality in the Region of Murcia from 4 August 2018 to 9 August 2018 and, subsequently, between 10 August 2018 and 16 August 2018, in another municipality in the province of Cadiz from where they travelled to various points in the province. Two of them usually resided in the Region of Murcia, and after their stay in Cadiz, they returned to their community. The third one resided in Madrid and after his stay in Cadiz, he travelled to a town in Malaga where he stayed from 16 August 2018 to 25 August 2018. On 25 August 2018, he stayed overnight at the family’s holiday home in Murcia and returned to Madrid on 26 August 2018. All three presented clinical signs compatible with dengue and progressed favourably. The case in Madrid required hospital admission [8].

The two cases residing in the Region of Murcia started symptoms on 18 August 2018 and 23 August 2018, a man and a woman aged 59 and 78 years, respectively. The first serology was performed on 19 September 2018, and IgM and IgG antibodies to dengue were detected. Diagnostic confirmation was performed by the National Microbiology Centre (CNM), by PCR with samples taken during the acute period of the disease in the first case and by neutralization techniques in the second case [8].

The Madrid case, a 39-year-old man, started showing symptoms on 27 August 2018. He was admitted to a private hospital in the Community of Madrid on 4 September 2018. A blood sample from 7 September 2018 tested positive for dengue by PCR in a private laboratory. Given the results of the Murcia cases, the CNM, as the National Reference Laboratory, requested this sample for confirmation, which was positive by PCR and detection of dengue antigen [8].

Analysis of the two sequences obtained by PCR revealed that the infection was due to dengue virus serotype 1 [8, 21]. Considering the date of onset of symptoms of the three cases and the incubation period of the disease, the place of acquisition of the infection could not be established with certainty [8, 21].

At the end of October, two new cases of autochthonous dengue were confirmed, possibly associated with the first cases, and in November, an additional case was reported with no apparent link to the previous cases [8].

On 26 October 2018, the CNM confirmed two new cases of dengue virus infection in two males aged 19 and 53, who were related to each other and resided in Murcia and who had not travelled outside that autonomous community in the 15 days prior to the onset of symptoms. Symptoms began on September 27 and 30, respectively. Both cases were confirmed at the CNM by PCR and by detection of dengue NS1 antigen [8, 21].

The 59-year-old case resident in Murcia from the first cluster, who had initiated symptoms on 18 August 2018, was on 11 September 2018, in the locality of residence of the latter two cases [8, 21]. Genetic sequencing analysis (identical in four cases: the men from Madrid and Murcia from the first cluster and the two from the second cluster) suggested that it could be the same virus [8, 21].

On 15 November 2018, the CNM confirmed by NS1 antigen detection another case of dengue virus infection in a young male resident in Catalonia who had started symptoms compatible with dengue on 17 October 2018 and who had not travelled outside his municipality of residence in the incubation period of the disease [8, 21].

Between 2019 and 2021, there were a total of 623 cases of dengue in Spain, of which 428 (69%) were confirmed [50]. Two autochthonous cases were reported in 2019 and none in 2020 or 2021 [50]. The first autochthonous case was detected in Madrid transmitted through sexual intercourse [51]. At the end of September 2019, two confirmed cases of dengue were reported in two men residing in the municipality of Madrid, in which epidemiological and microbiological investigations determined one imported and one autochthonous case, the latter most likely acquired through sexual contact [51].

On 20 September 2019, the CCAES was notified of a case of dengue with no history of travel outside Spain in the previous two months [51]. The case was a 36-year-old male who presented to the emergency department on 18 September 2019, with symptoms suggestive of the disease. On 15 September 2019, he presented with fever, headache, myalgia, dorsalgia, diarrhoea, and rash. He underwent a rapid test that was positive for dengue virus AgNS1 antigen, as well as serological determination of IgM and PCR (positive), but IgG negative. As he did not require hospitalization, he was sent home with symptomatic treatment and, on suspicion of being an autochthonous case, the serum sample was sent to the CNM, where it was confirmed by a second PCR technique [51].

The cohabiting partner of the case was a 41-year-old male, who reported the onset of very similar symptoms on 5 September 2019. Serum and urine samples were collected, and only the urine sample was PCR positive. This result was also confirmed at the CNM on 19 September 2019. This case had been in Cuba from 28 August 2019 to 30 August 2019, and in Dominican Republic between 2 September 2019 and 4 September 2019, and was therefore considered to be an imported case [51].

Once notification of the cases was received, the response actions foreseen in both the surveillance protocols of the National Epidemiological Surveillance Network and the Vector-borne Disease Preparedness and Response Plan were initiated [51].

Entomological investigations at the home of the cases and in the vicinity of the house were negative for *Ae. albopictus*, the mosquito vector of the disease present in Spain. Another municipality in the Community of Madrid where the cases had been on 31 August 2019 was also inspected, with no evidence of the presence of *Aedes* spp. All this was consistent with the data provided by the entomological surveillance carried out in the Community of Madrid since 2017, which only detected a stable presence of *Ae. albopictus* in the municipality of Velilla de San Antonio, an area far from the places visited by the cases [51].

In both cases, partial sequencing of the virus and subsequent phylogenetic analysis revealed that it was DENV-1 and that the viral sequence obtained was identical in the two patients, coinciding with that already detected in other cases imported from Cuba and analysed at the CNM [51].

The cases had unprotected sex within three days of the onset of symptoms in the imported case, so semen samples from both patients were requested and tested positive for dengue virus by PCR. Genetic sequencing of this sample showed identical results to the other samples studied. In the absence of data supporting possible vector-borne transmission and in the absence of other less common routes, the sexual route was considered the most likely route of transmission in the autochthonous case [51]. Nevertheless, it is important to note that sexual transmission in Spain occurred in a single case and was an exceptional event.

In 2022, there were a total of 503 cases of dengue, of which 358 (71.2%) were confirmed, none of them autochthonous [52]. However, on 1 February 2023, Germany reported two cases of dengue (one confirmed and one probable), along with four cases compatible with epidemiological link, in residents of Germany with a history of travel to the Balearic Islands in August 2022, specifically to Ibiza, during the incubation period [45, 46].

In 2023, there were a total of 615 cases, of which 398 (64.7%) were confirmed [53]. There were three autochthonous cases, all confirmed and reported by Catalonia, involving residents of the province of Barcelona. There were two women and one man, all under the age of 45. Two of the cases required hospitalization, and all progressed favourably with full recovery [53].

On 5 September 2023, the health authorities of Catalonia reported a case of autochthonous dengue fever. The case was a 12-year-old male, resident in Barcelona, with no history of travel to endemic areas, who stayed in the province of Tarragona intermittently between 25 July 2023 and 24 August 2023. On 22 August 2023, he started symptoms compatible with dengue virus infection. On 4 September 2023, a weakly positive PCR result for dengue was obtained, which was confirmed on 14 September 2023, as serotype DENV-1 [53].

On 20 October 2023, the health authorities reported the second case of autochthonous dengue during that year. The case was a 25-year-old woman, resident in Barcelona, with no history of travel to endemic areas. On 29 September 2023, she developed symptoms consistent with dengue virus infection. On 6 October 2023, she tested positive for IgM (IgG and PCR negative). She presented seroconversion on 16 October 2023 (IgM and IgG positive) and PCR positive in the urine sample. In addition, samples were sent to the National Reference Laboratory [53].

On 27 October 2023, the health authorities reported the third case of autochthonous dengue fever during 2023 in Catalonia. The case was a 43-year-old woman, resident in the province of Barcelona, with no history of travel to endemic areas. On 2 October 2023, she developed symptoms compatible with dengue virus infection. On 24 October 2023, she tested positive for IgM and positive for IgG; serum PCR was negative, and urine PCR was weak positive [53].

In 2024, there were a total of 1,119 cases, of which 788 (70.4%) were confirmed [54]. There were six autochthonous cases, five women and one man, all under the age of 35 [54]. Five of the cases shared the same address and workplace, and the address of a sixth case was very close to theirs. In addition, France reported two confirmed cases, with probable exposure in the same area. Of the six cases detected in Spain, two were hospitalized and later discharged to their homes, two cases had mild symptoms and did not require hospitalization, and two other cases were asymptomatic (see Table 5).

**Table 5.** Summary of dengue cases in Spain

Location	Dates/interval	Cases	Source
Spain – Region of Murcia & Cádiz (family cluster)	Exposures 4–16 Aug 2018; symptom onset 18–27 Aug 2018	3 autochthonous cases (same family)	[8]
Spain – Murcia (second cluster, related cases)	Symptom onset 27 & 30 Sep 2018; confirmed 26 Oct 2018	2 autochthonous cases (PCR/NS1 confirmed)	[8, 15]
Spain – Catalonia (single case)	Symptom onset 17 Oct 2018; confirmed 15 Nov 2018	1 autochthonous case (NS1 confirmed)	[8, 15]
Spain – Madrid (sexual transmission)	Symptoms 5–15 Sep 2019; confirmations 19 Sep–Oct 2019	1 autochthonous case (sexual transmission; cohabiting partner imported)	[46]
Spain – Catalonia (Barcelona province)	Onsets 22 Aug–2 Oct 2023; notifications 5 Sep–27 Oct 2023	3 autochthonous confirmed cases (2 hospitalized; all recovered)	[49–52]
Spain – Single area (same address/workplace; region not specified)	2024 season	6 autochthonous cases (2 hospitalized; 2 mild; 2 asymptomatic)	[53]

### Surveillance protocols and health responses in Spain

The detection of dengue cases in Madeira in 2012 made it necessary to assess the risk of reintroduction of the *Ae. aegypti* vector in Spain [33]. In October 2012, *Ae. aegypti* had not been detected since its eradication in Spain in 1953. However, *Ae. Albopictus* was detected in Catalonia in 2004 and spread along the Mediterranean coast [33].

Imported cases of dengue were reported in both Andalusia and Catalonia in 2018. Relevant control measures were taken, but the presence of the virus in local vectors could not be demonstrated. In the absence of travel and other risk factors, vector-borne transmission via *Ae. albopictus* was the most likely transmission hypothesis. Microbiological studies of the viral strains, together with epidemiological data, suggested that transmission occurred from two index cases imported from areas with active transmission [21].

The organization of the response to the detection of imported cases in Spain differs depending on whether they are in areas with or without the presence of a competent vector [5]. In areas with competent vectors, an active search for cases around the residence of each imported case, which continues for 45 days after the last reported case, is conducted. Autochthonous cases are reported urgently to the CCAES of the MISAN, which notifies the WHO under the International Health Regulations 2005 (IHR-2005) [55] and the EWRS as it is a disease with an impact on public health, which is unusual and poses a risk of international spread.

Furthermore, if an autochthonous case is confirmed in the territory or local transmission is detected, the response is intensified, and all sectors of the community become involved in actions for the prevention and control of this disease: education, health, environment, tourism, infrastructure, etc. Health education of the community in these areas is crucial to prevent transmission, focusing both on individual behaviour to avoid bites and on collective behaviour to ensure good environmental management.

Entomological surveillance serves operational purposes, to determine the presence of competent vectors, assess changes in the geographical distribution of vectors, obtain information on parameters that help estimate vector capacity, evaluate the resistance of insecticides used in control programmes, obtain information on the parameters that influence the development of vectors, assess the effectiveness of control measures, and facilitate appropriate and timely decision-making for each situation [56–58]

In Spain, vector-control responsibilities largely fall to municipal authorities. In autonomous communities where *Ae. albopictus* has become established, such as Catalonia and the Balearic Islands, and in others, where there are other native mosquito species, strategies are being developed to combat these vectors.

At the national level, the MISAN has been running an entomological surveillance programme since 2008. The two fundamental objectives are (1) the detection of imported vectors competent for the transmission of exotic infectious diseases, focusing on the main ports and airports in Spain, and especially vectors with implications for public health; and (2) conducting surveillance in areas with potential for the expansion of *Ae. albopictus* to detect recently colonized populations. Entomological surveillance of imported vectors focuses on two complementary aspects: the capture of adult mosquitoes, with the aim of broadening the spectrum of species that can be captured, thus enabling the early detection of imported species; and the active search for larvae to detect the possible establishment of these exotic species.

Once notification of the first three autochthonous cases in Spain was received on 4 October 2018, the three Autonomous Communities involved (Andalusia, Madrid, and Murcia) and the MISAN coordinated the response actions provided for in the surveillance protocols of the National Epidemiological Surveillance Network and in the Preparedness and Response Plan for vector-borne diseases. The autonomous communities involved conducted an epidemiological investigation of the three cases to identify the periods and places of possible exposure to the vector. An active prospective and retrospective search for cases was also carried out in all municipalities of interest. In this regard, communication and sensitization of health professionals at the healthcare and laboratory levels were reinforced to ensure early detection of cases and timely reporting. An entomological investigation was also carried out in the areas where the cases were hosted, both in the province of Cadiz and in the Region of Murcia [8, 21].

These same activities were implemented after the second family cluster detected in Murcia, as well as after the detection of the recent case in Catalonia, as established in the Protocol for Prevention and control of mosquito-borne arbovirosis in Catalonia, where on the same day of the confirmation of the case, entomological inspection was carried out in the home and surroundings with negative results for the capture of female *Ae. albopictus.* From the data obtained, it was deduced that the population of this vector was very low at that time [8, 21]. The MISAN and the Murcia Regional Ministry of Health maintained permanent contact to establish coordinated actions in the follow-up of the cases. Epidemiological and entomological investigations were carried out around the detected cases, and vector-control measures were applied in the areas of possible exposure.

The measures taken by the public health authorities and the decrease in mosquito density at the time of the year meant that the probability of autochthonous transmission at that time was considered very low.

According to the *Aedes* surveillance programme carried out in all municipalities in the Region of Murcia, there is evidence of *Ae. albopictus* presence in 2018 in 31 of its 45 municipalities, including the one in which the first three cases occurred in August. At the specific site of the holiday accommodation, environmental samples were collected on 8 October 2018 in two locations after microbiological confirmation of the cases. Six *Ae. albopictus* larvae were detected and tested for the presence of dengue virus by real-time PCR, with negative results. Subsequently, samples collected in ovitraps and traps in the environment of the first cluster were analysed, as well as samples collected in the environment of the cases of the second cluster, by different sampling techniques. In all four areas of possible exposure of the cases, initially or in their viraemic period, 46 immature forms and nine adults (females) of *Ae. albopictus* were studied and were also negative for the presence of dengue virus [8, 21].

During 2018, a single case of imported dengue was reported in the province of Cádiz. This was a male from Cuba who started symptoms on 8 August 2018, resident in a municipality located about 50 km from the hotel where the family stayed. Epidemiological investigations did not identify any link between this imported case and the three autochthonous cases in the first cluster. Nor was any link established with the other imported cases reported in Andalusia during that year [8].

In Murcia, no imported dengue cases were diagnosed in 2018. An active search and review of the medical records of all travellers with negative dengue serology requested in differential diagnosis processes was performed; in any case, the extraction could have been performed in the first days of illness and be a false negative; and no compatible situation was found [8, 21]. In Catalonia, in addition to the autochthonous case, 52 imported cases of dengue were confirmed in 2018 [8, 21].

In 2019, one autochthonous case was detected in Madrid, transmitted through sexual intercourse [51]. This case is the first in which it was concluded after investigation that transmission occurred through sexual transmission, taking into account that it was detected in an area without the presence of mosquito vectors of the disease and that there was no other possible route of exposure in the case [51, 59]. Although this event demonstrated that the dengue virus is capable of sexual transmission, this is an infrequent route of low epidemiological relevance, so the population-level risk from this event was considered very low [51]. It was considered advisable to revise the management recommendations in view of the appearance of new autochthonous and imported cases, which until then had focused on the prevention of mosquito vector bites and advice on blood products and transplants and did not include the prevention of sexual transmission [51].

After receiving a report from Germany of dengue cases that had spent time in Ibiza in 2022, the authorities of the Balearic Islands took action, and the National Epidemiology Centre and the Epidemiology Service of the Balearic Islands identified the existence of a probable index case, imported from Mexico and usually residing in Madrid, who started symptoms on 11 August 2022 and stayed in Ibiza between 11 August 2022 and 31 August 2022, in the same locality as the cases detected by Germany [36]. An environmental epidemiological assessment survey was carried out on the case. According to his statement, during the entire period of viraemia – between 11 August 2022 and 18 August 2022 – and until 20 August 2022, he remained at his home on the island, travelling only to the medical centre where he was treated, located in the city of Ibiza. The home was a single-family house with land, without vegetation, of recent construction. The case stated that he did not perceive the presence of mosquitoes in his home, and he was not bitten by mosquitoes because he controlled this possibility during the time he remained in his home. The technicians carried out the environmental assessment, obtaining climatological data on maximum, average, and minimum temperature, the environment of the house, and the activities carried out by the case. After evaluating the different variables established and taking into account that the case had adopted individual protection measures, it was concluded that the risk was moderate, so it was decided that it was not necessary to use adulticide treatments *in situ.* Recommendations were made to implement entomological surveillance, eliminate breeding sites, and inform the public [45].

The stay of the second imported case in Ibiza during its viraemic period in the same location as the autochthonous cases detected by Germany, and with sufficient time for the extrinsic incubation period in the mosquito, supported the theory that this case was the index case of the outbreak described and that transmission was carried out through the bite of *Ae. albopictus* in the locality where all the identified cases coincided [45].

After learning of the detection of autochthonous cases in Ibiza, in February 2023, the Balearic Islands authorities scheduled an entomological inspection prior to the start of the season (first half of March) and convened a meeting of the Communicable Diseases Committee, which included technicians from the Environmental Health Service, the Blood and Tissue Bank, and the Ministry of Agriculture. Another meeting was scheduled prior to the start of the vector activity season with the Consell Insular de Ibiza and the island’s five town councils, for the implementation of strict vector surveillance and control plans. In Ibiza, the period of the greatest activity of *Ae. albopictus* is between May and November, a season that coincides with the maximum influx of tourists to the island [45].

In September 2024, an outbreak of indigenous dengue transmission was investigated in an area of Tarragona, Catalonia. The Catalan Public Health Agency (ASPCAT) reported the following actions: active search for new cases through inspections to detect possible sources of transmission from mosquitoes and alerting primary care and hospital services in the area to detect suspected cases. In addition, work was carried out directly at the municipal level to provide recommendations for insect control and protection against mosquito bites. The Blood and Tissue Bank carried out systematic screening for dengue and other mosquito-borne diseases in donors in Catalonia [60].

### Risk of future transmission in Spain

The risk level of future dengue transmission in Spain is defined by the physical, social, economic, and environmental conditions or processes that increase the percentage of the population susceptible to and the probability of exposure to the virus.

First, globalization and the movement of people and goods increase the probability of exposure to dengue, given the likelihood of the dengue virus being introduced into Spain. The risk of introduction exists throughout the country through imported cases spread across the territory and the introduction of imported vectors at points of entry and by land in other parts of the territory [5].

Autochthonous transmission of vector-associated diseases depends on the presence and density of the vector, the introduction of the virus by an infected traveller from endemic areas or by an infected mosquito, the presence of a population susceptible to infection, the coincidence in space and time of an imported viraemic case with the vector, and the possibility that both virus and vector find the conditions favourable for transmission [61].

Furthermore, sociodemographic and cultural factors influence the interaction between the vector and humans. As described above, the dengue vector has a strong preference for humans, on whom it feeds preferentially. It is also a mosquito that is active during the day and in peridomestic areas. The culture of the Spanish population and the climate favour the rate of contact, as people spend more time outside their homes. In addition, the growth of cities and new single-family housing developments on the outskirts have created new breeding grounds for mosquitoes, increasing their population and favouring contact with humans [5].

At this time, the existence and establishment of the vector on the Mediterranean coast have been confirmed, although there is a risk of the vector spreading to other areas with climatic and environmental conditions conducive to its development through land communication. In addition, there is a risk of the vector being introduced into new locations through international maritime, land, and air traffic from countries where this vector exists [5].

The establishment of *Ae. albopictus* mainly on the Mediterranean coast, together with international travel and importation of cases, means that the risk of autochthonous cases cannot be ruled out [52].

The probability of sporadic autochthonous cases occurring in areas where *Ae. albopictus* is present during periods of high vector activity (May–November) is considered moderate and very low at other times of the year, especially from October onwards [45]. In the rest of the territory, where the vector is not present, it is non-existent [8, 21].

Epidemic dynamics are strongly influenced by variation in the timing of dengue virus introduction, seasonal temperature, transmission rates, mortality rate, and mosquito population [34, 35]. The transmission of vector-borne diseases, such as dengue fever, could be influenced by global climate change. Climate change in Europe has led to an increase of 0.8 °C over the last 100 years, parallel to global climate change, mainly softening winters, especially in the north of the continent. Climate change predictions in Spain point to rainier and warmer winters, followed by hotter and drier summers. If this trend continues, it is possible that the high vector mortality during winters will decrease. The increase in temperature in Spain would increase the periods of vector activity, lengthening the seasons during which transmission would be possible. It would also increase the number of mosquito breeding sites, with a possible geographical spread and increase in the exposed population [62].

One of the key tools in the prevention and control of this disease is vector-control actions aimed at reducing the density of these mosquitoes. Among the main problems that have arisen in vector-control activities are insecticide resistance and the lack of effectiveness of these activities. The collaboration of the population in peridomestic vector control is key to preventing and controlling the spread of mosquitoes. Finally, the scarcity of human and material resources could hinder the implementation of vector-control programmes [34].

Dengue could become an emerging disease in Spain. The symptoms are similar to those of influenza, with some more specific signs and symptoms. Given the characteristics of the disease, in which most cases are mild and self-limiting, even asymptomatic, it is likely that some imported cases do not consult the health system and are therefore not diagnosed. It is therefore important that the entire population, and travellers in particular, are aware of the disease and the protective measures and that they visit a health centre if they have compatible symptoms on their return from their travels. On the other hand, health workers should take into account the possibility of this disease in the differential diagnosis of febrile processes, especially in travellers, but also in people with no history of travel who reside in areas colonized by the vector during the months of vector activity [8, 21]. Suspicion of this syndrome is not common among healthcare professionals, which will make detection of cases difficult, and late detection could favour infection of vectors. However, even with these limitations, the impact of the disease in Spain is considered very low, given that most cases would not develop into serious illness and that the National Health System is capable of detecting and managing cases correctly [8, 21, 45].

Our conclusions are based on a non-systematic synthesis of published reports and official surveillance summaries and may therefore be affected by reporting bias and incomplete ascertainment. Surveillance intensity, diagnostic capacity (including access to PCR/NS1/serology), and application of case definitions differ across European settings and over time, potentially leading to under- or over-estimation of risk when comparing jurisdictions. Moreover, EU-level case definitions – while harmonized – may be implemented unevenly, further complicating cross-country comparisons. Finally, local vector abundance, micro-climatic conditions, and public health response can vary within short distances, limiting generalizability beyond the specific places and periods considered in the available reports.

## Conclusions

Evidence from recent European experiences indicates that the potential for autochthonous transmission of dengue and other *Aedes*-borne diseases is context dependent and varies over time and space. Autochthonous events in Europe have increased in frequency in the last decade, but remain highly focal and heterogeneous across settings, reflecting differences in vector presence and density, the timing and intensity of virus introductions through infected travellers or (rarely) infected mosquitoes, and local eco-climatic suitability for onward transmission. These patterns are further shaped by variability in surveillance intensity, diagnostic capacity, and case ascertainment practices across countries, which can influence detection and reporting of cases.

In Spain, areas with established *A. albopictus* warrant heightened vigilance during months of peak vector activity. While the available data are compatible with a low overall observed impact to date – given the small numbers and generally mild clinical presentations – this inference should be interpreted with caution because true risk is conditional on timely introductions, local vector ecology, and the sensitivity of surveillance systems. Consequently, sporadic autochthonous cases should be expected in receptive areas during periods of high vector activity, whereas sustained transmission remains uncertain and will likely depend on seasonal and micro-environmental conditions as well as public health response capacity.

From a preventive standpoint, rapid identification and isolation of imported viraemic cases – particularly when coordinated with integrated vector management – remain plausible strategies to reduce the probability of onward transmission; however, their effectiveness in European settings will depend on operational feasibility, community uptake, and ecological suitability and should be evaluated locally. Harmonized EU case definitions provide a framework to standardize classification and reporting, yet differences in implementation persist across jurisdictions and may affect cross-country comparisons of risk.

Overall, our conclusions emphasize uncertainty: current observations are consistent with low to moderate observed risk of limited autochthonous dengue transmission in receptive Spanish territories, but substantial uncertainty remains due to incomplete and non-uniform data streams and the inherently stochastic nature of introductions and focal transmission. Strengthening timely detection, ensuring access to confirmatory diagnostics, and maintaining adaptive vector-control capacity are prudent priorities to mitigate episodic transmission while improving risk estimates over time.

## Data Availability

All data analysed in this review are publicly available from third-party sources, and all underlying datasets can be accessed by other researchers. No new primary data were generated as part of this review.

## References

[1] World Health Organization. *Dengue - Global situation.* 2024; https://www.who.int/emergencies/disease-outbreak-news/item/2024-DON518 (accessed 7 February 2025).

[2] World Health Organization. *Dengue.* 2025; https://www.who.int/news-room/fact-sheets/detail/dengue-and-severe-dengue (accessed 29 September 2025).

[3] Lim A, et al. (2025) The overlapping global distribution of dengue, chikungunya, Zika and yellow fever. Nature Communications. 16, 3418. 10.1038/s41467-025-58609-5 PMC11986131 40210848

[4] Bhatt S, et al. (2013) The global distribution and burden of dengue. Nature 496, 504–507. 23563266 10.1038/nature12060 PMC3651993

[5] Centro de Coordinación de Alertas y Emergencias Sanitarias, et al. Evaluación del Riesgo de Introducción y Circulación del Virus de Dengue en España. 2013; https://www.sanidad.gob.es/areas/alertasEmergenciasSanitarias/situacionRiesgo/docs/evRiDe_5_13.pdf (accessed 7 February 2025).

[6] Gossner CM, et al. (2022) Dengue virus infections among European travellers, 2015 to 2019. Eurosurveillance. 27, 2001937. 35027102 10.2807/1560-7917.ES.2022.27.2.2001937 PMC8759115

[7] Herrero-Martínez JM, Sánchez-Ledesma M and Ramos-Rincón JM (2023) Dengue importado y autóctono en España. Revista Clínica Española. 223, 510–519. 37507047 10.1016/j.rceng.2023.07.007

[8] Centro de Coordinación de Alertas y Emergencias Sanitarias, et al. Primeros casos de dengue autóctono en España Actualización noviembre 2018. 2018; https://www.sanidad.gob.es/profesionales/saludPublica/ccayes/alertasActual/docs/ERR_Dengue_autoctono_Espana.pdf (accessed 7 February 2025).

[9] European Centre for Disease Prevention and Control. Autochthonous cases of dengue in Spain and France, 1 October 2019; https://www.ecdc.europa.eu/sites/default/files/documents/RRA-dengue-in-Spain-France_1Oct2019.pdf (accessed 7 February 2025).

[10] Brem J, et al. (2023) Dengue “homegrown” in Europe (2022 to 2023). New Microbes and New Infections 56, 101205. 38094104 10.1016/j.nmni.2023.101205 PMC10715994

[11] Lambrechts L, Scott TW and Gubler DJ (2010) Consequences of the expanding global distribution of Aedes albopictus for dengue virus transmission. PLOS Neglected Tropical Diseases 4, e646. 20520794 10.1371/journal.pntd.0000646 PMC2876112

[12] Richards SL, Anderson SL and Alto BW (2012) Vector competence of Aedes aegypti and Aedes albopictus (Diptera: Culicidae) for dengue virus in the Florida Keys. Journal of Medical Entomology 49, 942–946. 22897056 10.1603/me11293

[13] Kamgang B, et al. (2019) Risk of dengue in Central Africa: Vector competence studies with Aedes aegypti and Aedes albopictus (Diptera: Culicidae) populations and dengue 2 virus. PLOS Neglected Tropical Diseases. 13, e0007985. 31887138 10.1371/journal.pntd.0007985 PMC6953884

[14] Whitehorn J, et al. (2015) Comparative susceptibility of Aedes albopictus and Aedes aegypti to dengue virus infection after feeding on blood of viremic humans: Implications for public health. The Journal of Infectious Diseases. 212, 1182–1190. 25784733 10.1093/infdis/jiv173 PMC4577038

[15] Angel B and Joshi V (2008) Distribution and seasonality of vertically transmitted dengue viruses in Aedes mosquitoes in arid and semi-arid areas of Rajasthan, India. Journal of Vector Borne Diseases. 45, 56–59. 18399318

[16] Fortuna C, et al. (2024) Assessing the risk of dengue virus local transmission: Study on vector competence of Italian Aedes albopictus. Viruses 16, 176. 38399952 10.3390/v16020176 PMC10893310

[17] Cevidanes A, et al. (2023) Invasive Aedes mosquitoes in an urban—peri-urban gradient in northern Spain: evidence of the wide distribution of Aedes japonicus. Parasites and Vectors. 16, 1–10. 37452412 10.1186/s13071-023-05862-6 PMC10349466

[18] Artigas P, et al. (2021) Aedes albopictus diversity and relationships in south-western Europe and Brazil by rDNA/mtDNA and phenotypic analyses: ITS-2, a useful marker for spread studies. Parasites and Vectors. 14, 1–23. 34174940 10.1186/s13071-021-04829-9 PMC8235640

[19] Centro de Coordinación de Alertas y Emergencias Sanitarias, et al. Riesgo de detección de nuevos casos autóctonos de enfermedades transmitidas por Aedes en España. 2024; https://www.sanidad.gob.es/areas/alertasEmergenciasSanitarias/alertasActuales/dengue/docs/20240619_ERR_EnfermTransmitidasAedes.pdf (accessed 7 February 2025).

[20] Centro de Coordinación de Alertas y Emergencias Sanitarias, et al. Riesgo de aparición de nuevos casos autóctonos de enfermedades transmitidas por Aedes en España. 2023; https://www.sanidad.gob.es/areas/alertasEmergenciasSanitarias/alertasActuales/dengue/docs/ERR_EnfermTransmitidasAedes_05072023.pdf (accessed 7 February 2025).

[21] Centro de Coordinación de Alertas y Emergencias Sanitarias, et al. Dengue autóctono en España. 2^a^ actualización. 2019; https://www.sanidad.gob.es/areas/alertasEmergenciasSanitarias/alertasActuales/dengue/docs/ERR_Dengue_autoctono_mayo2019.pdf (accessed 7 February 2025).

[22] European Commission. Commission Implementing Decision (EU) 2018/945 of 22 June 2018 on the communicable diseases and related special health issues to be covered by epidemiological surveillance as well as relevant case definitions. 2018; https://eur-lex.europa.eu/eli/dec_impl/2018/945/oj/eng (accessed 7 February 2025).

[23] Gjenero-Margan I, et al. (2011) Autochthonous dengue fever in Croatia, August–September 2010. Eurosurveillance. 16, 19805. 21392489

[24] La Ruche G, et al. (2010) First two autochthonous dengue virus infections in metropolitan France. Eurosurveillance. 15, 19676. 20929659

[25] European Centre for Disease Prevention and Control. Clusters of autochthonous Chikungunya cases in Italy. 2017; https://www.ecdc.europa.eu/sites/default/files/documents/RRA-chikungunya-Italy-update-9-Oct-2017.pdf (accessed 13 March 2025).

[26] Succo T, et al. (2016) Autochthonous dengue outbreak in Nîmes, South of France, July to September 2015. Eurosurveillance. 21, 30240. 10.2807/1560-7917.ES.2016.21.21.30240 27254729

[27] Franke F, et al. (2019) Autochthonous chikungunya and dengue fever outbreak in Mainland France, 2010-2018. European Journal of Public Health. 29, 616–617.

[28] European Centre for Disease Prevention and Control. Dengue outbreak in Madeira, Portugal. 2013; https://www.ecdc.europa.eu/sites/default/files/media/en/publications/Publications/dengue-madeira-ECDC-mission-2013.pdf (accessed 17 March 2025).

[29] Wilder-Smith A, et al. (2014) The 2012 dengue outbreak in Madeira: Exploring the origins. Eurosurveillance. 19, 20718. 24602277 10.2807/1560-7917.es2014.19.8.20718

[30] European Centre for Disease Prevention and Control. Dengue outbreak in Réunion, France. 2018; https://www.ecdc.europa.eu/sites/default/files/documents/Dengue%20outbreak%20in%20Reunion,%20France.pdf (accessed 13 March 2025).

[31] Vincent M, et al. (2023) From dengue outbreaks to endemicity: Reunion Island, France, 2018 to 2021. Eurosurveillance. 28, 2200769. 37470738 10.2807/1560-7917.ES.2023.28.29.2200769 PMC10360367

[32] Centro de Coordinación de Alertas y Emergencias Sanitarias, et al. Actualización del Brote de Dengue en la Isla de Madeira, Portugal. 2012; https://www.sanidad.gob.es/areas/alertasEmergenciasSanitarias/alertasActuales/dengue/docs/actDen27_11.pdf (accessed 17 March 2025).

[33] Centro de Coordinación de Alertas y Emergencias Sanitarias, et al. Detección de Brote de Dengue Autóctono en la Isla De Madeira, Portugal. 2012; https://www.sanidad.gob.es/areas/alertasEmergenciasSanitarias/alertasActuales/dengue/docs/broDenMad.pdf (accessed 17 March 2025).

[34] Lourenço J and Recker M (2014) The 2012 Madeira dengue outbreak: Epidemiological determinants and future epidemic potential. PLOS Neglected Tropical Diseases. 8, e3083. 25144749 10.1371/journal.pntd.0003083 PMC4140668

[35] Salami D, et al. (2020) Simulation models of dengue transmission in Funchal, Madeira Island: Influence of seasonality. PLOS Neglected Tropical Diseases. 14, e0008679. 33017443 10.1371/journal.pntd.0008679 PMC7561266

[36] European Centre for Disease Prevention and Control. Seasonal surveillance of dengue in the EU/EEA, weekly report. 2025; https://www.ecdc.europa.eu/en/dengue/surveillance-and-updates/seasonal-surveillance-dengue-eueea-weekly (accessed 17 March 2025).

[37] Direção Regional da Saúde, Autoridade de Saúde Regional. Vigilância da Dengue e do *Aedes Aegypti* na RAM. 2025; https://www.madeira.gov.pt/drs/pesquisar/ctl/ReadInformcao/mid/12350/InformacaoId/225572/UnidadeOrganicaId/9/LiveSearch/dengue (accessed 17 March 2025).

[38] Santé publique France. Surveillance des infections par les virus de la dengue, du chikungunya et du zika en France métropolitaine : données de l’année 2022. 2022; https://www.santepubliquefrance.fr/maladies-et-traumatismes/maladies-a-transmission-vectorielle/chikungunya/articles/donnees-en-france-metropolitaine/chikungunya-dengue-et-zika-donnees-de-la-surveillance-renforcee-en-france-metropolitaine-en-2022 (accessed 11 April 2025).

[39] World Health Organization. Dengue – Réunion, France. 2018; https://www.who.int/emergencies/disease-outbreak-news/item/01-may-2018-dengue-reunion-en (accessed 11 April 2025).

[40] Santé publique France. Chikungunya, dengue et zika - Données de la surveillance renforcée en 2019. 2019; https://www.santepubliquefrance.fr/maladies-et-traumatismes/maladies-a-transmission-vectorielle/chikungunya/articles/donnees-en-france-metropolitaine (accessed 11 April 2025).

[41] Santé publique France. Chikungunya, dengue et Zika - Données de la surveillance renforcée en 2020. 2020; https://www.santepubliquefrance.fr/maladies-et-traumatismes/maladies-a-transmission-vectorielle/chikungunya/articles/donnees-en-france-metropolitaine (accessed 11 April 2025).

[42] Santé publique France. Surveillance des infections par les virus de la dengue, du chikungunya et du zika en France métropolitaine : données de l’année 2021. 2023; https://www.santepubliquefrance.fr/maladies-et-traumatismes/maladies-a-transmission-vectorielle/chikungunya/articles/donnees-en-france-metropolitaine/chikungunya-dengue-et-zika-donnees-de-la-surveillance-renforcee-en-france-metropolitaine-en-2021 (accessed 11 April 2025).

[43] Santé publique France. Surveillance des infections par les virus de la dengue, du chikungunya et du zika en France métropolitaine : données de l’année 2023. 2024; https://www.santepubliquefrance.fr/maladies-et-traumatismes/maladies-a-transmission-vectorielle/chikungunya/documents/rapport-synthese/surveillance-des-infections-par-les-virus-de-la-dengue-du-chikungunya-et-du-zika-en-france-metropolitaine-donnees-de-l-annee-2023 (accessed 11 April 2025).

[44] Santé publique France. Chikungunya, dengue et zika - Données de la surveillance renforcée en France hexagonale 2024. 2024; https://www.santepubliquefrance.fr/maladies-et-traumatismes/maladies-a-transmission-vectorielle/chikungunya/articles/donnees-en-france-metropolitaine/chikungunya-dengue-et-zika-donnees-de-la-surveillance-renforcee-en-france-hexagonale-2024 (accessed 11 April 2025).

[45] Centro de Coordinación de Alertas y Emergencias Sanitarias, et al. Agrupación de casos de dengue autóctono en Ibiza. 2023; https://www.sanidad.gob.es/areas/alertasEmergenciasSanitarias/alertasActuales/dengue/docs/20230228_ERR_Dengue_autoctono.pdf (accessed 19 May 2025).

[46] García-San-Miguel L, et al. (2024) Detection of dengue in German tourists returning from Ibiza, Spain, related to an autochthonous outbreak, August to October 2022. Eurosurveillance. 29, 2300296. 38577804 10.2807/1560-7917.ES.2024.29.14.2300296 PMC11004590

[47] Istituto Superiore di Sanità. Febbre dengue. 2026; https://www.epicentro.iss.it/febbre-dengue/ (accessed 21 May 2025).

[48] Sacco C, et al. (2024) Autochthonous dengue outbreak in Marche Region, Central Italy, August to October 2024. Eurosurveillance. 29, 2400713. 39574388 10.2807/1560-7917.ES.2024.29.47.2400713 PMC11583309

[49] Monge S, et al. (2020) Characterization of the first autochthonous dengue outbreak in Spain (August–September 2018). Acta Tropica. 205, 105402. 32088276 10.1016/j.actatropica.2020.105402

[50] Centro Nacional de Epidemiología. Informe epidemiológico sobre la situación de dengue en España. Años 2019, 2020 y 2021. 2022; https://cne.isciii.es/documents/d/cne/informe_renave_dengue-202019-2021-pdf (accessed 22 February 2025).

[51] Centro de Coordinación de Alertas y Emergencias Sanitarias, et al. Transmisión sexual del virus dengue en España. 2019; https://www.sanidad.gob.es/profesionales/saludPublica/ccayes/Doc.Eventos/ERR_Dengue_FINAL.pdf (accessed 22 February 2025).

[52] Fernández-Martínez B and Díaz-García O (2023) Estudio epidemiológico del dengue en España, año 2022. Boletín Epidemiológico Semanal 4, 226–234.

[53] Centro Nacional de Epidemiología. Informe epidemiológico sobre la situación de dengue en España. Año 2023. 2024; https://cne.isciii.es/documents/d/cne/informe_renave_dengue-2023 (accessed 22 February 2025).

[54] Centro Nacional de Epidemiología. Informe epidemiológico sobre la situación de dengue en España. Año 2024. 2025; https://cne.isciii.es/documents/d/cne/informe_renave_dengue-2024 (accessed 22 February 2025).

[55] World Health Organization. International Health Regulations (2005) 3rd ed. 2016; https://www.who.int/publications/i/item/9789241580496 (accessed 7 February 2025).

[56] Abbasi E (2025) Aedes aegypti and dengue: insights into transmission dynamics and viral lifecycle. Epidemiology & Infection 153, e88. 40747604 10.1017/S0950268825100320 PMC12345063

[57] Abbasi E (2025) Climate Change and Vector-Borne Disease Transmission: The Role of Insect Behavioral and Physiological Adaptations. Integrative Organismal Biology 7, obaf011. 40330693 10.1093/iob/obaf011 PMC12053451

[58] Abbasi E (2025) Emerging and transboundary arboviral diseases: the role of insect vectors and key drivers such as climate change and urbanization. International Journal of Tropical Insect Science 45, 2833–2842.

[59] Liew CH (2020) The first case of sexual transmission of dengue in Spain. Journal of Travel Medicine 27, taz087. 31776571 10.1093/jtm/taz087

[60] Agència de Salut Pública de Catalunya. Actualització 16.09.2024 del brot de dengue autòcton a Catalunya. 2024; https://salutpublica.gencat.cat/ca/detalls/Article/dengue-actualitzacio (accessed 22 February 2025).

[61] Centro de Coordinación de Alertas y Emergencias Sanitarias, et al. Identificación del mosquito Aedes aegypti en Santa Cruz de Tenerife. 2023; https://www.sanidad.gob.es/areas/alertasEmergenciasSanitarias/alertasActuales/vectores/docs/20230206_Ae_aegypti_ERR.pdf (accessed 22 February 2025).

[62] López-Vélez R and Moreno RM (2005) Climate change in Spain and risk of infectious and parasitic diseases transmitted by arthropods and rodents. Revista Espanola de Salud Publica 79, 177–190. 15913053 10.1590/s1135-57272005000200006

